# Secundum Type Atrial Septal Defect in Patients with Trisomy 21—Therapeutic Strategies, Outcome, and Survival: A Nationwide Study of the German National Registry for Congenital Heart Defects

**DOI:** 10.3390/jcm10173807

**Published:** 2021-08-25

**Authors:** Astrid E. Lammers, Julia Stegger, Marc-André Koerten, Paul C. Helm, Ulrike M. Bauer, Helmut Baumgartner, Anselm S. Uebing

**Affiliations:** 1 Department of Cardiology III—Adult Congenital and Valvular Heart Disease, University Hospital Muenster, 48149 Münster, Germany; koerten@kompetenznetz-ahf.de (M.-A.K.); helmut.baumgartner@ukmuenster.de (H.B.); 2 Department of Paediatric Cardiology, University Hospital Muenster, 48149 Münster, Germany; julia.stegger@ukmuenster.de; 3 National Register for Congenital Heart Defects, 13353 Berlin, Germany; helm@kompetenznetz-ahf.de (P.C.H.); ubauer@kompetenznetz-ahf.de (U.M.B.); 4 DZHK (German Centre for Cardiovascular Research), 10785 Berlin, Germany; 5 Klinik für Angeborene Herzfehler und Kinderkardiologie, Universitätsklinikum Schleswig-Holstein, 24105 Kiel, Germany; uebing@pedcard.uni-kiel.de

**Keywords:** Trisomy 21, Down syndrome, atrial septal defect, pulmonary vascular disease, pulmonary hypertension, survival

## Abstract

(1) Secundum type atrial septal defect (ASD II) is usually considered a relatively benign cardiac lesion amenable to elective closure at preschool age. Patients with trisomy 21 (T21), however, are known to have a higher susceptibility for pulmonary vascular disease (PVD). Therefore, T21 children may present with clinical symptoms earlier than those without associated anomalies. In addition, early PVD may even preclude closure in selected T21 patients. (2) We performed a retrospective analysis of the German National Register for Congenital Heart Defects including T21 patients with associated isolated ASD II. We report incidence, demographics, therapeutic strategy, outcome, and survival of this cohort. (3) Of 46,628 patients included in the registry, 1549 (3.3%) had T21. Of these, 156 (49.4% female) had an isolated ASD II. Fifty-four patients (34.6%) underwent closure at 6.4 ± 9.9 years of age. Over a cumulative follow-up (FU) of 1148 patient-years, (median 7.4 years), only one patient developed Eisenmenger syndrome and five patients died. Survival of T21 patients without PVD was not statistically different to age- and gender-matched controls from the normal population (*p* = 0.62), whereas children with uncorrected T21/ASD II (including patients with severe PVD, in whom ASD-closure was considered contraindicated) showed a significantly higher mortality. (4) The outcome of T21-patients with ASD II and without PVD is excellent. However, PVD, either precluding ASD-closure or development of progressive PVD after ASD-closure, is associated with significant mortality in this cohort. Thus T21 patients with ASD II who fulfill general criteria for closure and without PVD should be offered defect closure analogous to patients without T21.

## 1. Introduction

Congenital cardiac defects are a frequent comorbitidy in patients with Trisomy 21. Overall, patients with Down syndrome are reported to have an increased risk of developing congenital cardiac defects compared to non-Trisomy 21 individuals [1,2]. Secundum-type atrial septal defect (ASD II) is the third most common cardiac lesion in patients with Trisomy 21 accounting for 9% of lesions after complete atrioventricular septal defects (AVSD) (51%) and ventricular septal defects (VSD) (25%) in this cohort [1].

Children remain asymptomatic during infancy and childhood and diagnosis is often the result of a coincidental finding on screening echocardiography for a heart murmur [3]. Depending on the degree of right heart volume overload and strain, patients are usually scheduled for elective closure at preschool age [4,5]. Children with large ASD II, where size and/or anatomy preclude interventional closure and no clinical evidence for pulmonary hypertensive vascular disease would be referred for surgical closure without a hemodynamic evaluation/cardiac catheterization in most institutions prior to elective surgery [4].

Pulmonary vascular disease (PVD) with elevated pulmonary vascular resistance may occur in patients with longstanding ASD II in the third or fourth decade of life. Progress to Eisenmenger physiology with right-to-left shunting at atrial level has been described in historical cohorts (albeit late in life in most cases) but appears to be rare in contemporary patients [6,7] probably due to availability of timely repair in more recent years. Patients with Trisomy 21 are more susceptible developing pulmonary hypertension—even in the absence of cardiac malformation [2,7]. It has been found that also without structural heart disease, Trisomy 21 patients develop persistent pulmonary hypertension of the newborn more often (reported incidence 1.2–5.2%) compared to non-Trisomy 21 children (reported incidence 0.1%) [8]. This is consistent with the generally elevated predisposition for PVD and pulmonary vascular changes reported in the pulmonary vasculature of these patients [9,10]. In addition, children with Trisomy 21 have a higher incidence of obstructive sleep apnea, resulting in higher CO_2_ levels and thus leading to an increased pulmonary vascular resistance. Additional problems like muscular hypotonia (‘floppy larynx’) or hypertrophic tonsils may contribute to the same mechanism of CO_2_ retention, which can aggravate the progress and severity of pulmonary hypertension [2].

We aimed to analyze therapy, morbidity and survival of patients, with Trisomy 21 and ASD II with a special focus on the presence of (pre-existing or persisting) pulmonary hypertension/pulmonary vascular disease after the intervention/surgery.

## 2. Materials and Methods

This was a retrospective analysis of the German National Register for Congenital Heart Defects for patients with Trisomy 21 and an isolated ASD II including all patients enrolled with the condition until December 2015. Therefore, patients with complete atrioventricular septal defects (AVSDs), primum atrial septal defects (ASD I) and partial AVSDs were excluded. We report incidence, demographics, therapeutic strategy, outcome, morbidity, and survival of this cohort. A database search was performed based on ICD-10 codes and the International Pediatric and Congenital Cardiac Code as introduced by the International Society for Nomenclature of Pediatric and Congenital Heart Disease screening for relevant Trisomy 21 codes [11]. All retrieved records were manually confirmed to have Trisomy 21 by reviewing their anonymized files held in the Registry. Furthermore, information on patients’ characteristics, previous medical history, including interventions/operations and outcome was collected from the National Registry. Survival and time of death were retrieved by reviewing medical records and by direct inquiry with the local registration office for all patients. Approval by the relevant Ethics Committee was obtained.

### Statistical Methods

Descriptive data are shown in absolute numbers and percentages for categorical variables and medians as well as interquartile ranges (IQR, corresponding to the 25th and 75th percentile) for continuous variables. For statistical comparison between groups Mann–Whitney U tests or t-tests were used depending on the normality of the underlying data and chi squared tests were employed for categorical variables. Normal distribution was tested based on the results of the Shapiro–Wilk test and after visual inspection of Q-Q plots. Kaplan–Meier analyses were used to assess survival in this population and log-rank tests were used for comparison between groups. To compare survival relative to an age- and gender-matched normal population sample estimated standardized mortality ratios (SMRs) were employed. This is based data of the general German population, using the statistical method reported by Finkelstein et al. [12]. Life table data published by the Federal Statistical Office was used as a basis for the analysis. For all analyses, a two-tailed *p* < 0.05 was considered statistically significance. The statistical software R V.3.1.0 (R Foundation for Statistical Computing, Vienna Austria) was utilized for all analyses [13].

## 3. Results

**Figure 1:**
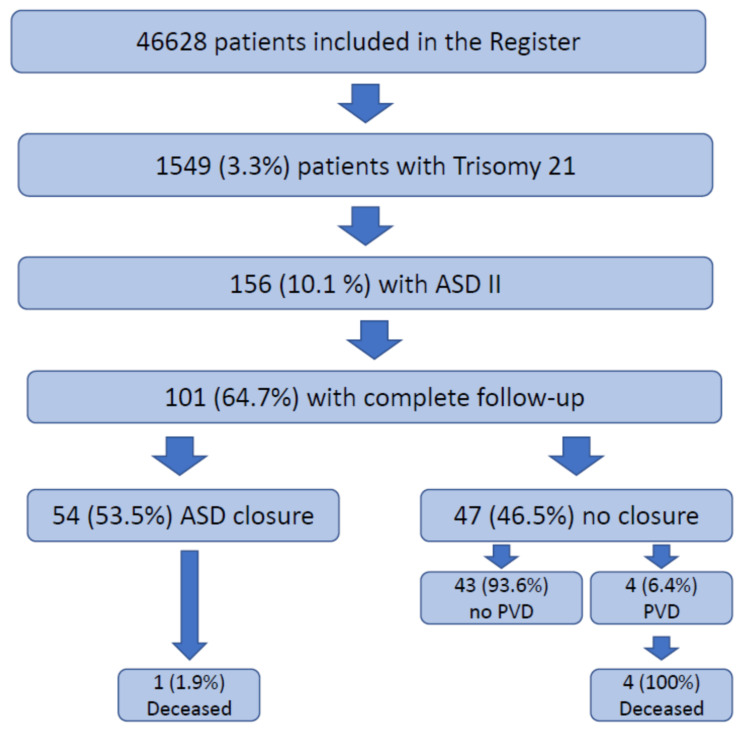
Flowchart illustrating the patient cohort and the outcome in the study population. ASD = atrial septal defect; PVD = pulmonary vascular disease.

Out of a total of 46,628 patients with congenital heart disease enlisted in the National Registry for Congenital Heart disease, 1549 (3.3%) patients had a confirmed diagnosis of Trisomy 21 (Figure 1). Of these patients, 156 (10.1%) were diagnosed with an ASD II. The mean age of ASD II patients at last contact was 12.1 ± 8.6 years, 77 patients (49.4%) were female and complete follow-up information was available in 101 patients. Fifty-four patients (53.5%) underwent closure of their ASD, whereas in 47 patients the ASD was left untreated. The mean age at first intervention/surgery was 6.4 ± 9.9 years. However, due to the skewed distribution of age at closure, the median (IQR) age at closure was 2.7 (0.8–7.8) years. Of the 54 patients in whom the decision was taken to close the ASD II, 22 children (40.7%) underwent interventional closure. The majority of children received an Amplatzer device (*n* = 18), while Figulla flex (*n* = 2), starflex (*n* = 1), helex (*n* = 1) and solysafe (*n* = 1) devices were also used occasionally. In one child the device dislocated after delivery and had to be retrieved surgically. This child thus underwent surgical closure of the ASD II subsequently. In total there were 32 patients (59.3%) who underwent surgical closure of the ASD. According to the documentation available, eight children had a direct closure (25% of the surgical cases), while in 24 patients (75% of the surgical cases) a patch-closure technique was used. In the group, in whom ASDs were closed, there were two patients each with persisting pulmonary hypertension in the interventional group and in the surgical group (patch closure), one of the latter patients died.

In the group with the untreated ASD II, the majority of patients (*n* = 43; 93.6%) did not develop documented pulmonary hypertension and there were either no signs of right ventricular volume overload and/or the shunt did not fulfil hemodynamic criteria for closure. In contrast, three patients (1.9%) were diagnosed with PVD and a conscious decision was made to leave the ASD II patent. All these patients died during the follow-up period. Only one patient (without any correction/intervention) developed Eisenmenger syndrome in our cohort at the age of 16 years.

Over a cumulative follow-up period of 1148.3 patient years (corresponding to a median follow-up 7.4 years), five patients (corresponding to 3.2% of the population) died. Overall survival in the whole cohort on actuarial analysis at 1, 10, 20 und 40 years of age was 97.8%, 96.8%, 93.3% and 77.8%, respectively. All patients with documented PVD (*n* = 3) or Eisenmenger syndrome (*n* = 1) died during follow-up. In addition, one patient died due to reasons unrelated to any cardiovascular/pulmonary vascular problem (accident). In the Eisenmenger patient who died at the age of 24 years, ASD II closure had been discussed and recommended repeatedly earlier in life but had been declined. One death of a patient with end-stage PH was procedure-associated; the patient decompensated and died during an attempt to place a Potts shunt. The other two deaths occurred in children with progressive PVD, one with significant pulmonary comorbidity (prematurity, acute respiratory distress syndrome and bronchopulmonary dysplasia). Notable, other diseases included hematological conditions with leukemia in two patients (1.3%), who are still alive at the end of follow-up.

**Figure 2:**
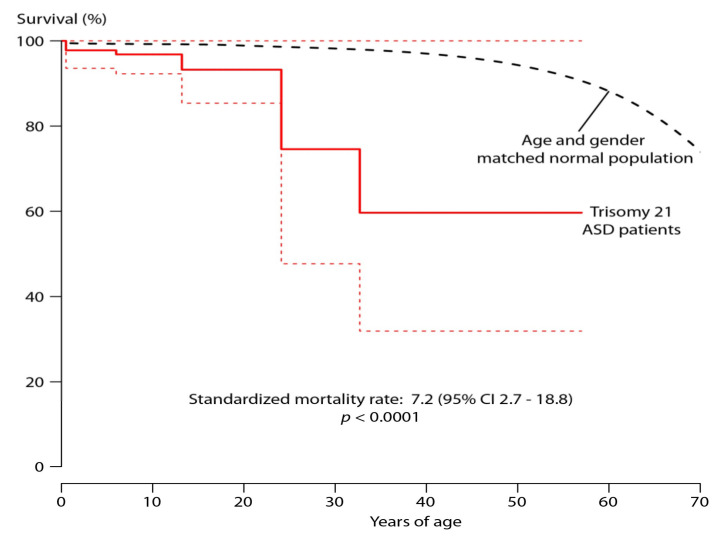
Survival of the overall Trisomy 21 ASD population (with 95% confidence intervals dotted line) compared to that of an age and gender matched sample of the general population. For details see Text. ASD refers specifically to ASD II.

**Figure 3:**
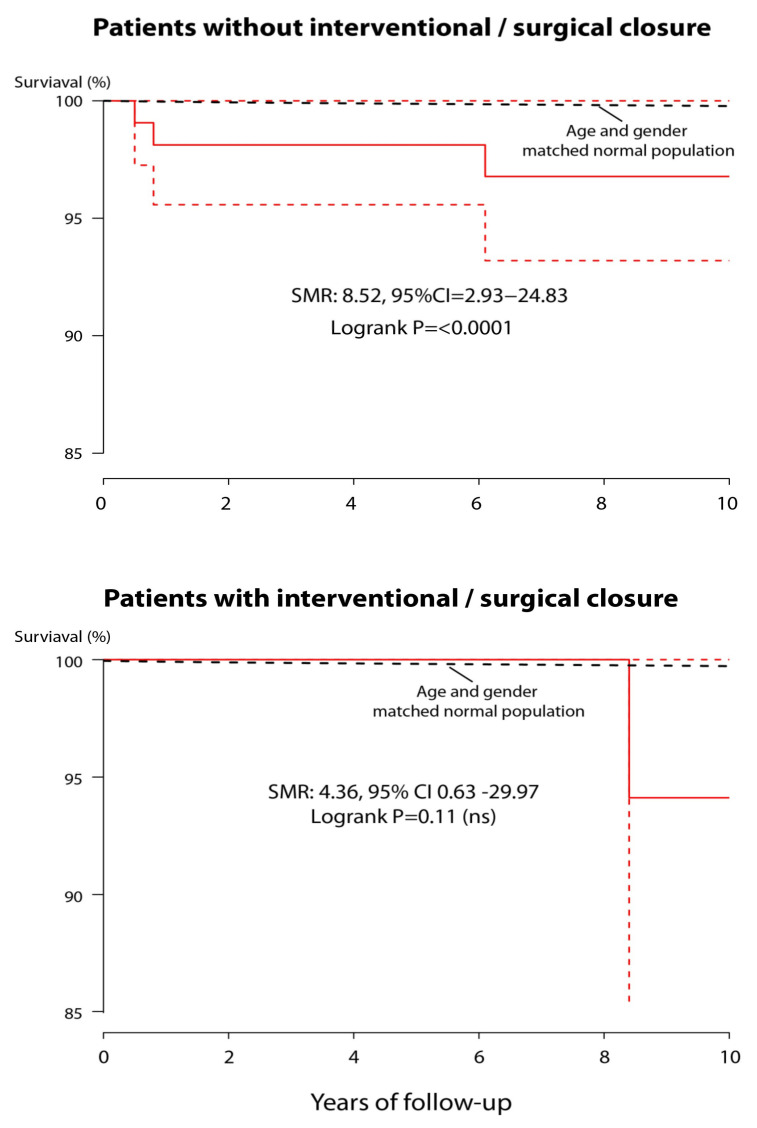
Survival of the Trisomy 21 ASD population (with 95% confidence intervals—dotted line) stratified by amenability to interventional or survival defect closure compared to that of an age and gender matched sample of the general population. For details see text. SMR = standardized mortality ratio. ASD refers specifically to ASD II.

Overall, survival of Trisomy 21 ASD II patients was significantly worse compared to an age- and gender-matched sample from the German population with a SMR of 7.2 (95% CI 2.7–18.8%, *p* < 0.0001) as illustrated in Figure 2. When assessing survival by type of treatment, however, patients after interventional/surgical closure were found to have a significantly better survival rate compared to patients without ASD II closure (*p* = 0.039). Actual survival of Trisomy 21 patients after ASD correction was not statistically different to that of an age- and gender-matched control group from the overall population (SMR 4.4; 95% CI 0.6–30.0; *p* = 0.11) on relative survival analysis, whereas uncorrected Trisomy 21/ASD II patients showed a significantly higher relative mortality. (SMR 8.5; 95% CI: 2.9–28.8; *p* < 0.0001) as shown in Figure 3. Specifically, for patients with diagnosed pulmonary hypertension SMR was 166.9 (95% CI 37.3–486.9; *p* < 0.0001).

## 4. Discussion

The current study illustrates outcome in patients with Trisomy 21 and a coexistent ASD II. The study by and large confirms the current strategy of offering eligible Trisomy 21 patients ASD II closure, liberally. Though Trisomy 21 patients are afflicted by additional comorbidities and general survival prospects (irrespective of cardiac conditions) maybe impaired [2], the current data supports the notion that Trisomy 21 ASD II after ASD closure have similar survival prospects compared to the general population. In contrast, our study also raises concerns in Trisomy 21 patients with pulmonary vascular disease. While Eisenmenger syndrome was only documented in one patient, pulmonary vascular disease emerged as a relevant problem and was associated with significantly elevated mortality. The decision of ASD closure in Trisomy 21 patients is complex and requires individual assessment. Beyond the usual considerations in ASD patients without Trisomy such as symptoms, right ventricular dilatation and pulmonary vascular resistance [3,4], additional considerations are relevant in Trisomy 21 patients. Especially a high level of suspicion of emerging PVD is required and a low threshold for invasive assessment is necessary.

Although a left-to-right shunt at atrial level is usually well tolerated in healthy infants, a subgroup of patients exists, in whom even a high pulmonary blood flow of a pre-tricuspid shunt is not well tolerated [5,14,15,16]. This particularly applies to patients with pulmonary compromise or comorbidities, and early closure should be considered in this situation. These patients often benefit from early closure and symptoms may alleviate after the shunt is closed. [17]. Furthermore, early closure of a defect with significant left-to-right shunt may prevent additional volume overload and damage to the pulmonary vasculature, preventing additional vascular remodeling and disease progression via sheer stress. In contrast, for patients with established PVD, ASD II closure may be detrimental and lead to acute right heart failure and increased mortality [18,19]. In fact, we have learned from patients with severe pulmonary vascular disease and/or Eisenmenger physiology that survival of those patients with PVD and a shunt communication (intra- or extracardiac) is superior to those with severe PVD and no possibility to shunt [19]. Creation of an atrial communication (atrial septostomy) or Pott’s shunt, therefore, nowadays presents an active treatment strategy—in addition to advanced drug therapies—for patients with end-stage pulmonary hypertension and no shunt [20,21]. Hence, in a child with possible progressive PVD, the presence of an atrial septal defect may be desirable to relieve right ventricular strain and maintain function, acting as a pop-off valve. This is beneficial, not only to maintain cardiac output, in the setting of acute increases in PVR, but also to restore ventricular geometry and improve ventriculo-ventricular interactions by diminishing diastolic septal shift into the left ventricle [20,21].

During an arguably short follow-up period, only one of the uncorrected patients with T21 and ASD II developed Eisenmenger syndrome. The mean age of surgery or intervention of patients with Trisomy 21 in current study was comparable with the timing of intervention of patients with an ASD II without any comorbidity [4]. In addition, overall survival data of those corrected were comparable to a normal population. From a management strategy point, this suggests that for most Trisomy 21 ASD patients similar strategies and recommendations for ASD II closure apply as for the general population. There may still be, however, a small cohort of Trisomy patients with early onset, malignant and progressive pulmonary vascular disease, who present early in life and in whom ASD closure may be contraindicated or fenestrated closure may be a desirable strategy. This may particularly apply to patients with additional comorbidities, affecting the development of pulmonary hypertension regardless of the underlying cardiac lesion (e.g., in prematurely born children with Trisomy 21 and bronchopulmonary dysplasia).

However, from case series the longevity of the atrial fenestration is not guaranteed and the communication may close spontaneously [22]. In contrast, for those patients who develop progressive pulmonary vascular disease later in life, a Potts’ shunt may be a suitable alternative, preserving right ventricular function and maintaining cardiac output at the expense of a lower systemic saturation to the lower part of the body [20,21].

The current study is consistent with a previous study illustrating that mortality in Trisomy 21 patients with post-tricuspid lesions is largely driven by PVD and Eisenmenger syndrome [23]. While Eisenmenger syndrome appears to occur rarely in ASD patients, PVD has shown to be similarly associated with poor prognosis in ASD II Trisomy 21 patients.

### Strengths and Limitations

The total follow-up time of the study was too short to expect a relevant number of patients presenting with Eisenmenger physiology, as this usually occurs in the third or fourth decade of life [3,7]. However, our data support the notion that even in Trisomy 21 patients an isolated ASD will rarely trigger Eisenmenger syndrome early in life. It is obviously unknown, if the survival of the four patients who died during the follow-up period would were affected by closure of the defect. Due to limited data in this retrospective analysis, insufficient data was available on preclosure hemodynamics in this cohort. Therefore, we cannot distinguish preclosure PVD from late onset PVD after defect closure. Despite efforts to acquire data on nocturnal hypoventilation or CO_2_ retention, this information was largely missing. Clinicians should remain vigilant as these comorbidities may be underestimated and present a relevant clinical dilemma. We did not assess pulmonary hypertension and survival in patients with ASD II but without Trisomy 21 included in the register as the current study was focused entirely on Trisomy 21 patients. However, previous studies have shown that survival prospects of corrected ASD II patients (without Trisomy 21) are excellent and survival is comparable to normal individuals [3,24].

## 5. Conclusions

The outcome of Trisomy 21 patients with an ASD II but without PVD is excellent. However, PVD, either precluding ASD-closure or development of progressive PVD after ASD-closure, is associated with significant mortality in this cohort. Patients with Trisomy 21 with ASD II who fulfill general criteria for ASD closure should be offered, ASD device or—if an interventional closure is not possible—surgical defect closure liberally in the absence of PVD as survival of these patients was similar to that of the general population in our study.

## Data Availability

All data obtained from the National Registry for Congenital Heart Disease including this project are available from the Registry according to the rules and regulation of the registry upon reasonable request.
